# Exploration of shared features of B cell receptor and T cell receptor repertoires reveals distinct clonotype clusters

**DOI:** 10.3389/fimmu.2022.1006136

**Published:** 2022-10-20

**Authors:** Sang Bin Hong, Yong-Won Shin, Ja Bin Hong, Sang Kun Lee, Buhm Han

**Affiliations:** ^1^ Department of Medicine, Seoul National University College of Medicine, Seoul, South Korea; ^2^ Center for Hospital Medicine, Department of Neurosurgery, Seoul National University Hospital, Seoul, South Korea; ^3^ Department of Neurology, Charité Universitätsmedizin Berlin, Berlin, Germany; ^4^ Department of Neurology, Seoul National University Hospital, Seoul, South Korea; ^5^ Department of Biomedical Sciences, Brain Korea 21 (BK21) Plus Biomedical Science Project, Seoul National University College of Medicine, Seoul, South Korea; ^6^ Interdisciplinary Program in Bioengineering, Seoul National University, Seoul, South Korea

**Keywords:** immune repertoire, B cell receptor, T cell receptor, immune diversity, immune memory

## Abstract

Although B cells and T cells are integral players of the adaptive immune system and act in co-dependent ways to orchestrate immune responses, existing methods to study the immune repertoire have largely focused on separate analyses of B cell receptor (BCR) and T cell receptor (TCR) repertoires. Based on our hypothesis that the shared history of immune exposures and the shared cellular machinery for recombination result in similarities between BCR and TCR repertoires in an individual, we examine any commonalities and interrelationships between BCR and TCR repertoires. We find that the BCR and TCR repertoires have covarying clonal architecture and diversity, and that the pattern of correlations appears to be altered in immune-mediated diseases. Furthermore, hierarchical clustering of public B and T cell clonotypes in both health and disease based on correlation of clonal proportion revealed distinct clusters of B and T cell clonotypes that exhibit increased sequence similarity, share motifs, and have distinct amino acid characteristics. Our findings point to common principles governing memory formation, recombination, and clonal expansion to antigens in B and T cells within an individual. A significant proportion of public BCR and TCR repertoire can be clustered into nonoverlapping and correlated clusters, suggesting a novel way of grouping B and T cell clonotypes.

## 1 Introduction

The increasing availability of B cell receptor (BCR) and T cell receptor (TCR) repertoires through recent developments in high-throughput sequencing (1–4) has highlighted a pressing need to be able to interpret the accumulated data to derive biologically and clinically meaningful conclusions. Significant progress has been made in methods of analyzing aspects of the immune repertoire, providing measures of clonality, diversity, VDJ gene usage, sequence motifs, and network characteristics (5). These methods, in turn, have led to discoveries of repertoire characteristics in normal aging (6, 7), and in diseases such as cancer (8, 9), autoimmune disorders (9, 10), and infections (11, 12). Existing methods have focused on separate analyses of BCR and TCR repertoires, however, and no study to date has attempted to discover existing parallels within an individual’s BCR and TCR repertoires.

BCR and TCR repertoires share cellular machinery such as recombinase to produce a diverse set of receptors and evolve through common formative events such as infections and exposure to diverse antigens. Both B and T cells act in concert to orchestrate effective adaptive immune responses and drive out pathogens. Maturation of B cells to produce more antigen-specific antibodies occurs in a T cell-dependent manner, and T cells in turn can expand clonally in response to antigens presented by antigen presenting cells including B cells. In addition, both B and T cells keep a record of immune exposures in their memory cell subset. We hypothesized that the shared history of antigen exposures and shared cellular tools for recombination of B and T cells, as well as the interdependent manner through which B and T cells operate, result in commonalities in B and T cell repertoires in an individual. We further tried to find out whether there exist specific B or T cell clonotypes whose presence in BCR and TCR repertoires are interdependent and what the defining characteristics of such clonotypes are.

With this hypothesis and question in mind, we jointly analyzed the clonal distribution, diversity, clonal overlap, and network characteristics of BCR and TCR repertoires in healthy individuals and individuals with disease, including COVID-19 and autoimmune hepatitis (AIH). We further extracted clusters of highly correlated BCR and TCRs to examine their distinctive features.

## 2 Materials and methods

### 2.1 Immune repertoire sequencing data

We searched for receptor sequence datasets with both BCR and TCR sequences available for each subject in public datasets provided by iReceptor (13) and European Nucleotide Archive (14). We found five studies with B and T cell repertoire sequencing data from peripheral blood of both healthy and individuals with disease (**Table 1**).

**Table 1:** Demographic and sequencing information on the studies with immune repertoire data from both B and T cells used for analysis.

	Author(Year)	Sequencing method, Data processing	N	Median age (range)	Gender (% female)	B/T average clonotype count (SD)
**Healthy** **(N = 152)**	Rubelt et al. (2016)	Immune repertoire sequencing of FACS-sorted B/T cells (RNA), VDJPipe, pRESTO, IMGT/HighV-QUEST	10	NA (22-27)	NA	20852 (13164) 24791 (8908)
	Schultheiss et al. (2021)	Bulk immune repertoire sequencing (gDNA), Mixcr	57	50 (23-86)	60	1610 (1300) 7314 (3667)
	Schultheiss et al. (2020)	Bulk immune repertoire sequencing (gDNA) Mixcr	37	NA	NA	2311 (1298) 5818 (3058)
	Wen et al. (2020)	10x chromium single-cell V(D)J enrichment (RNA) Cell Ranger vdj pipeline	5	55 (30-80)	60	951 (352) 5449 (1745)
	Joseph et al. (2022)	immunoPETE protocol from Roche Sequencing (gDNA) In-house pipeline	43	43 (25-71)	44	8394 (5643) 23412 (12193)
**DA** **(N = 133)**	Schultheiss et al. (2022)	Bulk immune repertoire sequencing (gDNA), Mixcr	54 (AIH)	54 (20-79)	80	1649 (1309) 6863 (5298)
	Schultheiss et al. (2021)	Bulk immune repertoire sequencing (gDNA), Mixcr	37 (COVID)	NA (20-79)	32	3980 (2556) 9197 (6460)
	Wen et al. (2020)	10x chromium single-cell V(D)J enrichment (RNA) Cell Ranger vdj pipeline	10 (COVID)	58 (30-80)	50	999 (587) 2250 (1282)
	Joseph et al. (2022)	immunoPETE protocol from Roche Sequencing (gDNA) In-house pipeline	32 (COVID)	60 (28-88)	38	10145 (10743) 22059 (15431)

DA, disease-associated.

AIH, autoimmune hepatitis.

NA, not available.

Two studies with 94 healthy controls and 91 patients (AIH: N=54, COVID-19: N=37) (15, 16) amplified rearranged immunoglobulin heavy chain (IGH) and T-cell receptor beta chain (TRB) regions of genomic DNA, sequenced these using Illumina MiSeq, and processed the reads using the MiXCR framework (17), resulting in data for an average of 2,203 unique B cell clonotypes and 7,250 unique T cell clonotypes for each individual. One study with 10 healthy controls (18) used RNA from FACS-sorted immune cells to sequence IGH and TRB regions. Sequences were acquired using Illumina MiSeq and then processed using VDJPipe and pRESTO toolkit. This study provided sequence information for 20,852 T and 24,791 B cell clonotypes per subject on average. The next study with 10 healthy controls and 5 COVID-19 patients (19) acquired sequence reads using Chromium Single-Cell V(D)J Enrichment kit and processed them using the Cell Ranger vdj pipeline. Individuals in this dataset had 983 T cell clonotypes and 3,250 B cell clonotypes on average.

We conducted replication and validation analyses on a separate study with 43 healthy controls and 32 COVID-19 patients (20). In this study, genomic DNA from peripheral blood mononuclear cells were isolated to generate immune receptor libraries using the immunoPETE protocol from Roche Sequencing Solutions, providing sequence information for an average of 22,834 T cell clonotypes and 9,141 B cell clonotypes per individual.

Individual demographic information was available only for COVID-19 patients in two studies (N=47) and the replication study (healthy N=43, COVID-19 N=32). For the main study, we restricted our analyses to productive full-length CDR3 amino acid sequences of TRB and IGH.

### 2.2 Clonality and diversity metrics

Metrics of clonality and diversity between pairs of individuals were gathered for each repertoire using the R package immunarch (21) (v0.5.4). Clonality indices included clonal volume and clonal proportions occupied by the most frequent and rarest clones in terms of proportion and in rank. More specifically, clonal volume refers to the total number of unique clonotypes, indicating the total number of unique CDR3 amino acid sequences found in each repertoire. The next clonality measures were selected to capture the frequency distribution of clonotypes within each repertoire, spanning the most abundant clonotypes and the rarest clonotypes. Top clone proportions measure the cumulative fraction of the repertoire occupied by the 100 clonotypes with the highest clonotype counts. Hyperexpanded clone proportions refer to the fraction of the repertoire that is occupied by clonotypes that have counts that exceed 1% of the total number of all clones within each individual repertoire. Similarly, small clone proportions are sums of the proportions of the clonotypes that each take up less than 0.001% of the repertoire space. Finally, rare clone proportions are proportions of the repertoire occupied by clones with clonal counts fewer than three.

We chose several diversity metrics to cover several aspects of diversity. These included metrics of species richness that rely more heavily on clonal volume, including the Chao1 coefficient, as well as metrics that incorporate species evenness, including Hill number with q=1 and q=2, Gini-Simpson index, and Gini coefficient. The Chao1 coefficient estimates true diversity by considering the number of singletons and doubletons in the repertoire. Hill number with q=1 is equal to the exponential of Shannon’s entropy index, while Hill number with q=2 is the inverse of Simpson’s concentration index. Gini-Simpson index reflects the probability of interspecific encounter and the Gini coefficient measures how inequal species frequencies are along a frequency distribution. We have provided a more detailed description of how each metric was calculated in the **Supplementary Note**.

All metrics were calculated for each BCR and TCR repertoire individually, and correlation between an individual’s BCR and TCR metrics measured separately for the healthy and disease-associated (DA) groups of each dataset. The overall correlation coefficients for all healthy datasets and all DA datasets were calculated by meta-analyzing Spearman’s rank correlation coefficients with the metacor function from the meta package (v5.5-0) in R (v4.0.12). Significance was evaluated with false discovery rate (FDR)-adjusted *P*-values. Since clonality and diversity metrics can be profoundly affected by sequencing depth and quality, we conducted additional analyses to assess for the influence of these factors by analyzing correlations using multiple downsampling to 6 different fixed repertoire sizes (200, 500, 1000, 2000, 5000, 8000). For two studies with 94 healthy controls and 91 patients (15, 16), fastq files of sequenced reads were available, from which we extracted quality control metrics. The proportion of high-quality reads (quality score > Q30) was used as a covariate in a multivariate regression model to separately test whether sequencing quality affected correlations between summary statistics.

### 2.3 Network analysis

To construct networks and derive their network metrics, we likewise randomly downsampled individual repertoires to 6 repertoire sizes (200, 500, 1000, 2000, 5000, 8000), and removed frequency information for each clonotype. Networks were constructed by counting each clonotype as a single node and connecting them to clonotypes within 3 Levenshtein distances (LDs). To construct these networks, we used the imnet package (22). Based on these constructed networks, we calculated 6 global network metrics: assortativity, average degree, average clustering coefficient, density, local and global efficiency.

Assortativity measures how similar the connections within the graph are in terms of the node and degree. The average degree within a network refers to the average number of connections each node has. The average clustering coefficient denotes how many triangles a node participates in on average, reflecting how tightly connected the nodes are. Density is related to average degree in that it reflects the number of all the edges in the graph compared to the total number of nodes. Local and global efficiency both measure the shortest path between nodes, where local efficiency reflects shortest path distances of pairs of nodes within subgraphs and global efficiency measures the shortest path distances of all pairs of nodes in the graph. A more detailed description of the metrics is provided in the **Supplementary Note**.

These metrics were calculated with networkX v2.8 (23). Correlation between the global network metrics of BCR and TCR repertoires was measured separately for each study, and then combined in a meta-analysis using the metacor function from the meta package in R.

### 2.4 Cell subtype-specific public clonotype analysis

This part of the analysis was done on data provided by Rubelt et al. (18) (N=10), because this dataset provided information on the cell subtype of origin for each clonotype. We applied the conventional definition of public clonotypes as the identical sequences of full-length CDR3 amino acids found in two or more individuals and extracted the sequence information of public clonotypes. For each of the 6 cell subtypes included in the dataset (naïve B cell, memory B cell, naïve CD4+ T cell, memory CD4+ T cell, naïve CD8+ T cell, memory CD8+ T cell), we pooled all clonotypes from different individuals together and selected the 50 most frequently occurring public clonotypes. Next, for each of the 50 clonotypes and for each cell subtype category, we calculated the clonal proportions – i.e. clonal proportion occupied in relation to the whole clonal space of the individual’s cell subtype – within each individual. We then measured the correlation between all pairs of selected most frequent public clonotypes $\left(\begin{array}{c} 300\\ 2 \end{array}\right)$ correlations) over the samples. In order to see whether there are differential patterns in correlation depending on the cell subtype status of the clonotype, we counted the clonotype pairs with high and significant correlation coefficients, defined as Pearson’s r values exceeding 0.8 and *P-*values less than 0.05, within all $\left(\begin{array}{c} 6\\ 2 \end{array}\right)$ comparison categories. We visually represented the result in a correlation heatmap.

### 2.5 Correlation-based clustering using public clonotype proportions

Using public clonotypes, we wanted to calculate correlations between T and B clonotype proportions and perform clustering. We defined 4 sets of public clonotypes, i.e. same CDR3 amino acid sequences found in two or more individuals, for each B and T cell clonotypes of healthy and individuals with disease. As in the previous analysis, for each public B and T cell clonotype, its proportion, i.e. clonal proportion occupied in proportion to the whole B or T clonal space of an individual, was calculated in all four datasets. We then calculated the correlation between the proportions of all B and T cell clonotype pairs across all individuals in each healthy and DA groups of each dataset. Thus, we obtained different correlation coefficients for each of the respective B×T cell clonotype pairs for 4 healthy and 3 DA datasets. The correlation coefficients were combined in a meta-analysis as described above.

We visually represented the correlation matrix in a hierarchically clustered heatmap using the heatmap function of R (v4.0.12). Hierarchical clustering was done with hclust function in base R (v.4.0.12). Briefly, rows and columns were clustered based on dissimilarity matrices. In order to select the clonotypes belonging to the identifiable clusters, we used the InteractiveComplexHeatmap (v.1.2.0) package in R.

### 2.6 Motif discovery, analysis of amino acid properties, and public receptor sequence database

Further analyses were done based on the clusters of B and T cell clonotypes with similar correlation patterns that were visually selected from the hierarchically clustered heatmap. The analysis entailed three main parts: 1) discovery of enriched motifs, 2) comparison of average LDs of the CDR3 amino acid sequences, and 3) comparison of amino acid physical properties, which included Kidera factors 1 to 10, charge, core, disorder, MJ energy, hydropathy, volume, and polarity (24).

To look for novel motifs enriched within the clustered B and T cell clonotypes, we used STREME v5.4.1 (25), searching for motifs with a minimum width of 3 and a maximum width of 10, under the *P-*value threshold of 0.05. Next, to compare within-cluster CDR3 amino acid sequence characteristics to those of all public clonotypes, we generated 10,000 randomly selected sets of public clonotypes (4×10,000 sets of each healthy and DA B and T cell clonotypes) of comparable size to the selected clusters. Average pair-wise LD for each clonotype pair within the clusters and within the randomly selected sets were calculated with the stringdist function in R. Averages of amino acid physical properties of each cluster and each random set were calculated with vdjtools v1.2.1 (24). The distribution of the average LDs and amino acid physical property metrics in the selected clusters were compared against the distribution of the metrics calculated from the 10,000 randomly selected sets for each healthy and DA B and T cell clonotypes.

### 2.7 Relationship of TCR clusters to public database of TCR sequences with known antigen

Next, we referred to a public database of TCR sequences with known antigens: McPAS-TCR (updated August 5, 2021) and VDJdb (release 2022.02.23) (26, 27). From these databases, we extracted the TCR beta CDR3 amino acid sequences of specific to 11 antigen categories that had more than 3000 sequences each. These antigen categories were: Candida, CMV, SARS-CoV-2, diabetes mellitus type 1, EBV), influenza virus, HIV, SLE, and *Mycobacterium tuberculosis*. For each antigen category, we calculated the fraction of matching T cell clonotypes within each healthy and DA clusters. We then calculated the proportional enrichment of matching antigen-specific sequences by comparing these fractions to underlying proportions of antigen-specific sequences in the total pooled public T cell clonotypes from healthy and DA datasets.

### 2.8 Validation of results from the primary analysis in an independent dataset

To replicate and validate our findings, we retraced our steps of the primary analysis using a replication dataset. We computed correlations between BCR and TCR repertoire summary statistics of clonality, diversity, and network metrics as detailed in sections 2.2 and 2.3. We further displayed the combined results of all datasets in a forest plot.

Finally, we wanted to see whether the B and T cell clonotypes within the correlated clusters also tended to be found simultaneously within an individual in the independent dataset. We calculated how many clonotypes belonging to each clonotype cluster the individual repertoires contained, as a fraction of the size of the clustered clonotype groups:


$$Fr_{B,i}^{m}=N_{B,i}^{m}/N_{B}^{m}\text{ and }Fr_{T,i}^{m}=N_{T,i}^{m}/N_{T}^{m}$$

where $N_{B}^{m}$ refers to the total count of B cell clonotypes belonging to cluster *m* , and $N_{B,i}^{m}$ refers to the count of B cell clonotypes belonging to cluster *m*, that was observed in individual *i*. For example, if cluster *m* contained 10 different B cell clonotypes and if individual *i* had 3 of those clonotypes, then $Fr_{B,i}^{m}$ would be 0.3. The fraction for T cell clonotypes ( $Fr_{T,i}^{m}$ ) was defined likewise. For B cell clonotypes, we counted clonotypes that were closely similar (within 2 LD) to the clustered B cell clonotype to be counted, as there were very few clonotypes that exactly matched the clustered B cell clonotypes.

Suppose that cluster *m* represents a true correlated cluster between a group of B clonotypes and a group of T clonotypes. Then, in an independent dataset, $Fr_{B,i}^{m}$ and $Fr_{T,i}^{m}$ will be associated. We tested whether the fraction of matching clonotypes of each B cell clonotype belonging to a cluster ( $Fr_{B,i}^{m}$ ) can explain the fraction of matching T cell clonotypes from the paired cluster ( $Fr_{T,i}^{m}$ ) in a linear regression model, using the individual’s total immune repertoire size as a covariate, as below:


$$Fr_{T,i}^{m}∼Fr_{B,i}^{m}+C_{i},$$

where *C_i_* refers to the total number of B and T cell clonotypes within the repertoire of individual *i.* Note that the samples used for this regression iterate over all possible *m* and all individuals in the replication dataset.

To simulate null hypothesis, the same model was tested for significance 100 times using random B and T clonotype clusters of the same sizes as the original clonotype clusters, to compare the distribution of slope estimates and *P*-values.

### 2.9 Statistical tests

To assess for the presence of correlation of the collected metrics between BCR and TCR repertoires across the collected datasets, we used Spearman’s rank correlation coefficients for each dataset separately and combined these from each dataset in a fixed effects inverse-variance weighted meta-analysis using the metacor function from the meta package in R. FDR-corrected *p*-values using the Benjamini-Hochberg method (28) and a threshold of 0.05 were used to assess significance.

Fisher’s exact test was used to determine the significance of the differences in the numbers of significantly and highly correlated most frequent public clonotypes in memory and naïve B and T cell subtypes using base R. To compare distributions of randomly sampled data with data from derived from selected clusters, we used the Kolmogorov-Smirnov test.

## 3 Results

We collected immune repertoire sequencing data for a total of 285 individuals, comprising 152 healthy individuals and 133 subjects with disease (79 COVID-19 patients and 54 autoimmune hepatitis patients) from 5 independent datasets (15, 16, 18–20). All studies from which the sequence information was gathered performed targeted immune receptor sequencing. Brief information about the datasets is presented in **Table 1**. We used data from 4 studies (N=210) to discover our primary findings and used data from one study (N=75) to replicate our primary findings.

### 3.1 BCR and TCR repertoires show correlated clonal architecture and diversity in healthy individuals and in individuals with disease

**Figure 1:**
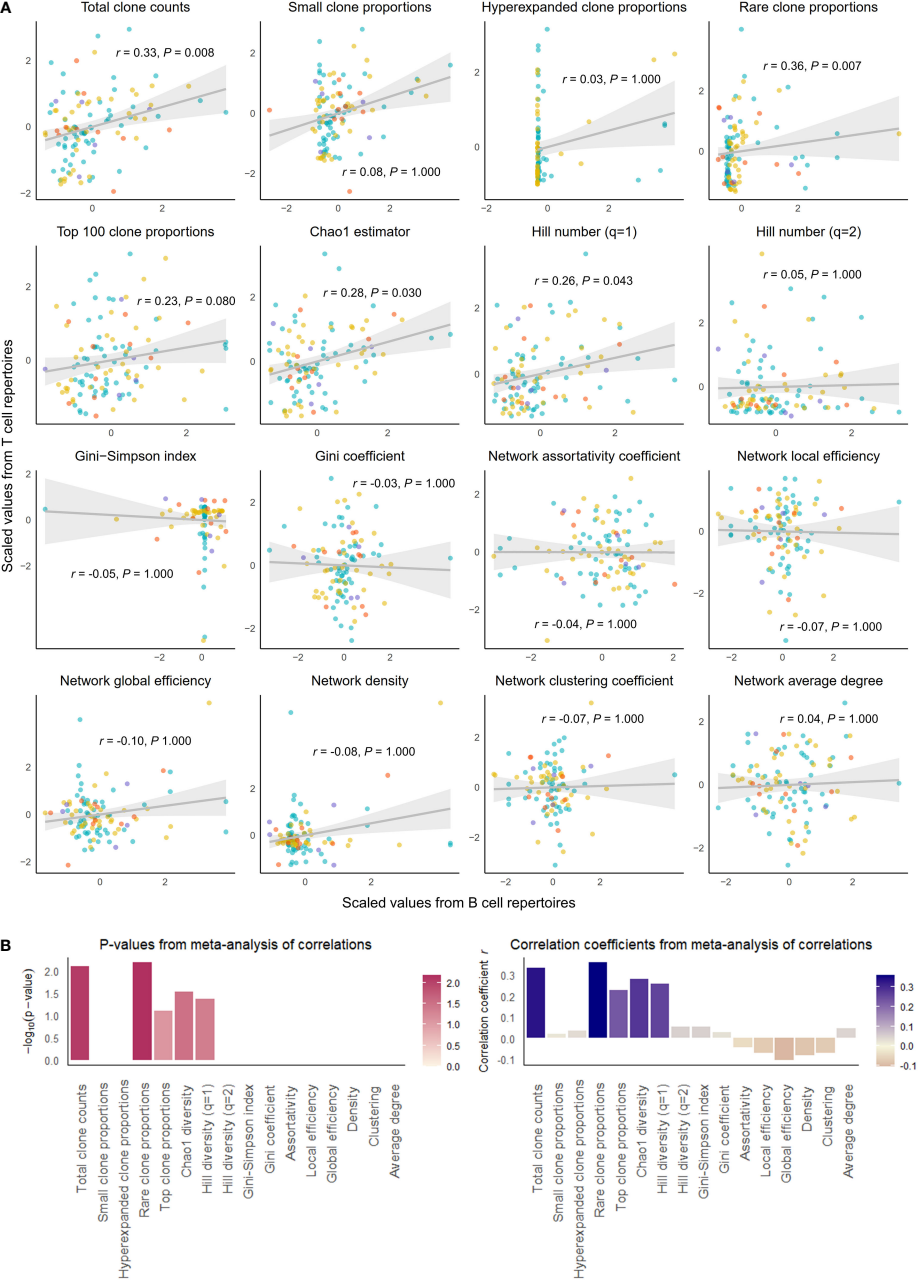
Correlation of select repertoire summary statistics between B and T cell repertoires of healthy individuals. **(A)** Scatterplot of scaled summary statistics for B and T cell repertoires of healthy individuals. Colors represent individuals from different studies. *r* and *P* values are derived from inverse variance-weighted fixed-effects meta-analysis across all studies **(B)** Bar plots of *P*-values (left) and correlations coefficients *r* (right) from the meta-analysis of correlations for different summary statistics.

**Figure 2:**
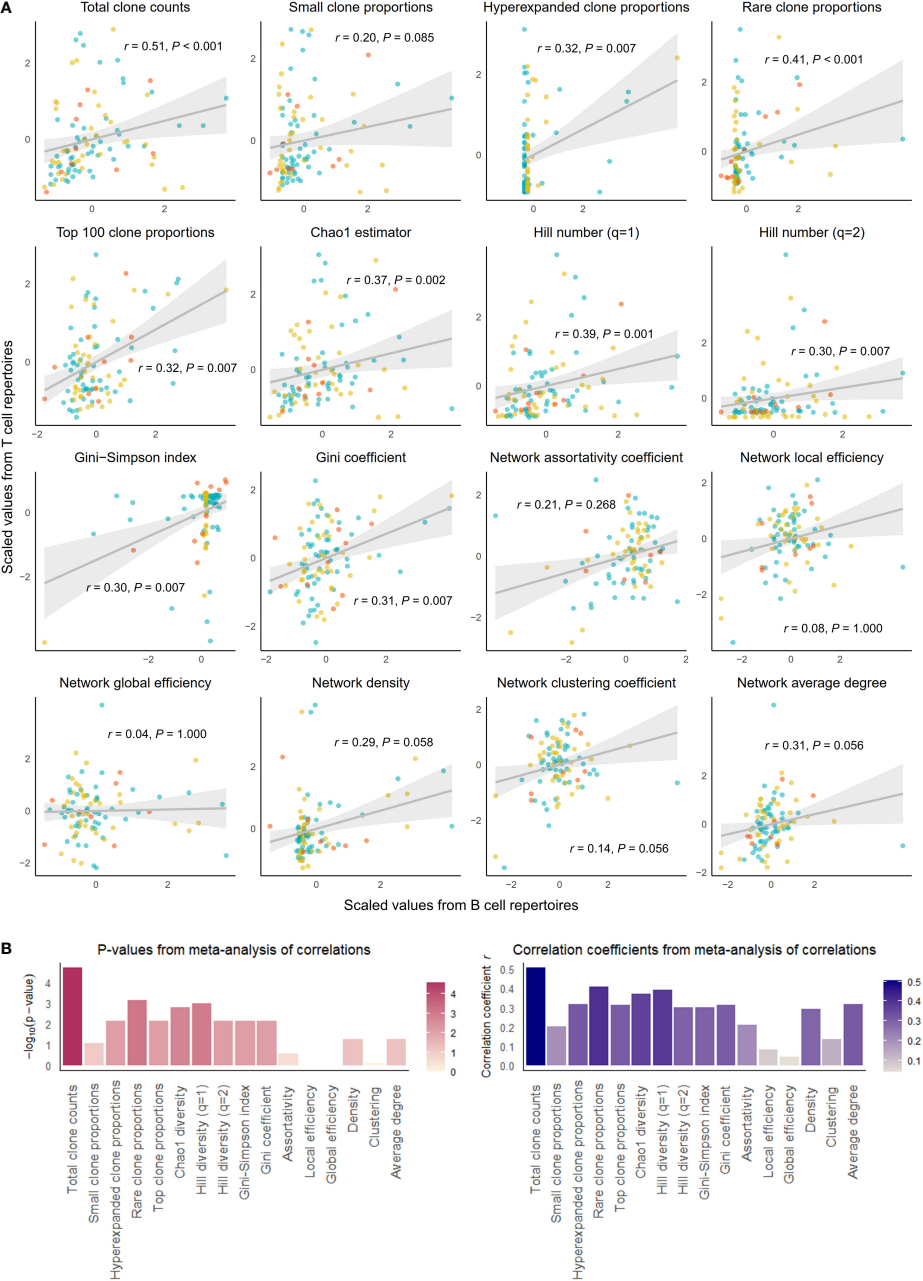
Correlation of select repertoire summary statistics between B and T cell repertoires of individuals with disease. **(A)** Scatterplot of scaled summary statistics for B and T cell repertoires of individuals with disease. Colors represent individuals from different studies. *r* and *P* values are derived from inverse variance-weighted fixed-effects meta-analysis across all studies **(B)** Bar plots of *P*-values (left) and correlations coefficients *r* (right) from the meta-analysis of correlations for different summary statistics.

To answer the question of whether clonal architecture of BCR and TCR repertoires are correlated within an individual, we tested four categories of summary statistics of clonal architecture for any correlation between BCR and TCR repertoires. These categories comprised (1) clonal volume, measured in the total number of unique clonotypes; (2) proportions of clonotypes belonging to each of following four different categories of clonal expansion, where the four categories were small clones (clones taking up less than 1/10000 of clonal space), hyperexpanded clones (clones taking up more than 1/10 of clonal space), rare clones (clones with clonal count of fewer than 3), and top 100 most frequent clones; (3) diversity metrics measuring species richness, including Chao1 estimator; (4) and diversity metrics incorporating abundance data, including Hill number with q =1 (equal to the exponential of Shannon’s entropy index), Hill number with q =2 (the inverse of Simson’s concentration index), Gini-Simpson index (the probability of interspecific encounter), and Gini coefficient quantifying inequality of clonotypes along a frequency distribution. **Figures 1**, **2** summarize the results.

We observed that clonal volumes were significantly correlated between the BCR and TCR repertoires in both healthy individuals (total unique clonotype count; *r =* 0.33, *P-*value = 0.001) and individuals with disease (*r =* 0.26, *P-*value = 0.028). This describes the tendency for any individual with a large number of BCR clonotypes to have a large number of TCR clonotypes.

The summary statistics describing proportions of selected clonotypes also showed strong correlations between the BCR and TCR repertoires within an individual, and notably, a number of summary statistics showed stronger correlations in disease than in health. Rare clone proportions were significantly correlated in both healthy and DA repertoires, but the correlation was greater in disease (*r =* 0.41, *P-*value < 0.001) than in health (*r =* 0.36, *P-*value = 0.007). Hyperexpanded clone proportions and top 100 clone proportions were significantly correlated in disease (*r =* 0.32, *P-*value = 0.007, *r =* 0.31, *P-*value< 0.007, respectively), whereas healthy individuals showed similar correlation directions without reaching significance (*r =* 0.03, *P*-value = 1.000, *r =* 0.23, *P-*value = 0.080, respectively).

In addition, the diversity metric measuring species richness was significantly correlated in both healthy individuals (Chao1 estimator: *r =* 0.28, *P-*value = 0.030) and disease subjects (Chao1 estimator: *r =* 0.37, *P-*value = 0.002). This pattern was also seen with Hill number with q=1 (R=0.26, *P-*value = 0.043 in healthy individuals, and R=0.39, *P-*value = 0.001 in individuals with disease). In contrast, several diversity metrics measuring species evenness showed significant correlations in disease (Gini-Simpson index: *r =* 0.30, *P-*value = 0.007, Gini coefficient: *r =* 0.31, *P-*value = 0.007) but not in health (Gini-Simpson index: *r =* 0.05, *P-*value = 1.000, Gini coefficient: *r =* 0.03, *P-*value = 1.000). Thus, overall, several summary statistics of clonal proportions and diversity showed weak to moderate correlations in both healthy and DA repertoires, indicating that there exist similarities in clonal structure and diversity within each individual’s BCR and TCR repertoires. These correlation patterns remained consistent when downsampled to multiple repertoire sizes and when quality control metrics inferred from fastq files were included as a covariate (**Supplementary Table 1**). Interestingly, the correlations of hyperexpanded, top, and rare clone proportions, as well as diversity metrics of species evenness, between BCR and TCR repertoires appear to be accentuated in the DA state compared to the healthy, suggesting that mobilization of the immune system to combat disease results in coordinated changes in both BCR and TCR repertoires that lead to increased similarities in the clonal architecture and diversity.

### 3.2 Network metrics from downsampled repertoires do not show clear correlation patterns between BCR and TCR repertoires

Next, we looked for correlations in network characteristics between BCR and TCR repertoires by calculating six global network metrics – assortativity coefficient, local and global efficiency, network density, network clustering coefficient, and average degree (See **Methods**). These metrics measure different characteristics and properties of network structures of BCR and TCR repertoires. Because the network metrics can greatly depend on the total number of clones, we uniformly subsampled BCR and TCR repertoires to multiple repertoire sizes (200, 500, 1000, 2000, 5000, 8000) from each individual repertoire to calculate the network metrics.

Unlike the tendency of consistent results regardless of repertoire size that was observed in the results of the correlations of clonality and diversity metrics, correlations between the network metrics of BCR and TCR repertoires varied with different subsampled repertoire sizes (**Supplementary Table 2**), such that consistent inferences about correlations of metrics between BCR and TCR repertoires could not be derived with the subsampled iterations. A reason for this inconsistency may be that the metrics are disproportionately affected by the inclusion of exclusion of clonotypes at the center of networks.

### 3.3 Analysis of correlation of the proportions of cell subtype-specific public clonotypes reveals increased high correlations between memory B and memory CD4+ T cells

In an effort to evaluate our initial hypothesis of whether shared immune memory of B and T cells manifests itself in their respective repertoires, we examined the correlation structure between naïve and memory immune cell subtypes with respect to their clonotypes. Among the 4 datasets we have been using so far, the Rubelt et al. (18) dataset (containing repertoire data from 10 healthy individuals) had information on which cell subtype each clonotype originated from, as they performed repertoire sequencing on FACS-sorted naïve and memory B and T cells. The dataset comprised two B cell types and four T cell types (naïve B, memory B, naïve CD4+ T, memory CD4+ T, naïve CD8+ T, memory CD8+ T). We defined the most frequently occurring (top 50) public clonotypes for each of the cell subtypes (50×6 clonotypes) and calculated each clonotype’s clonal proportion within the pool of their respective cell subtype. We then calculated the correlation between all pairs of selected clonotypes ( $\left(\begin{array}{c} 300\\ 2 \end{array}\right)$ correlations) over the samples. We wanted to see whether these correlations were distinctively high or low when correlation was measured between clonotypes of naïve and memory cell subtypes.

**Figure 3:**
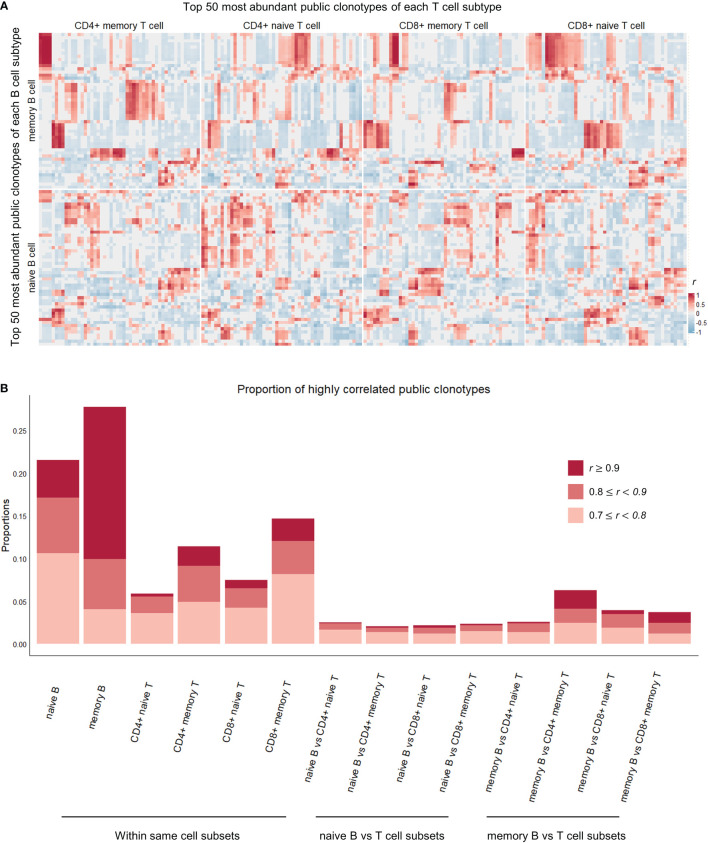
Correlation heatmap of top 50 most abundant public clonotypes and proportions of highly correlated clonotypes in each cell subtype comparison category. **(A)** Heatmap showing correlation coefficients *r* between the proportions of the top 50 most abundant B (vertical axis) and T (horizontal axis) public clonotypes of each naïve B, memory B, CD4+ naïve T, CD4+ memory T, CD8+ naïve T, CD8+ memory T cell subsets. The individual clonotypes have been hierarchically clustered based on the correlation coefficients within their respective cell subset comparison categories. The correlations between public clonotypes of memory B and memory T cells, in upper left square, are most pronounced and most clearly clustered, whereas correlations between naïve B cell public clonotypes and T cell public clonotypes from various T cell subsets are less pronounced. **(B)** Stacked boxplot showing the proportions of highly (*r* > 0.7) and significantly (*P*-value< 0.05) correlated public clonotypes within the same cell subsets (on the left), and between different B and T cell subsets (on the right).

We present the correlation results in **Figure 3**. As might be expected, a great number of significant high correlations (Pearson’s r greater than 0.7, *P-*value less than 0.05) were seen between clonotypes from the same cell subtype. For example, among public clonotypes of memory B cells, 11.8% of correlations were high and significant, whereas only 3.8% of correlations between public clonotypes of memory B cells and non-memory B cells were high and significant (Fisher’s exact test for proportions, Chi-squared statistic 165.21, *P-*value < 2.2e-16). This implies that the functional compartmentalization that is present within an immune cell subtype is revealed by our method of looking at the correlations between top public clonotypes.

Between B and T cell clonotypes of different cell subtypes, high and significant correlations were seen most frequently between memory B cell clonotypes and memory CD4+ T cell clonotypes. The proportion of high and significant correlations was significantly larger than the proportion of high and significant correlations between all B and T cell clonotypes (Fisher’s exact test for proportions, Chi-squared statistic 58.4, *P-*value = 2.151e-14, **Figure 3B**). This concentration of highly correlated clonotypes in memory B and memory CD4+ T cell subsets perhaps suggests of an increased interplay of immune functions and shared immune memory between memory B and memory CD4+ T cells.

More interestingly, public clonotypes from memory B cell and CD4+ memory T cell subsets displayed a tendency to cluster into well demarcated subsets (**Figure 3A**), whereas public clonotypes from naïve B cells and T cell subsets did not cluster into clear groups. Combined with the observation that memory B cells and CD4+ memory T cells have the highest proportion of public clonotypes that are highly correlated, this finding indicates that immune memory may be stored in these correlated clusters of public clonotypes of memory B and CD4+ memory T cells.

### 3.4 Distinct clonotype clusters revealed by hierarchical clustering of public clonotypes based on correlation of proportions

**Figure 4:**
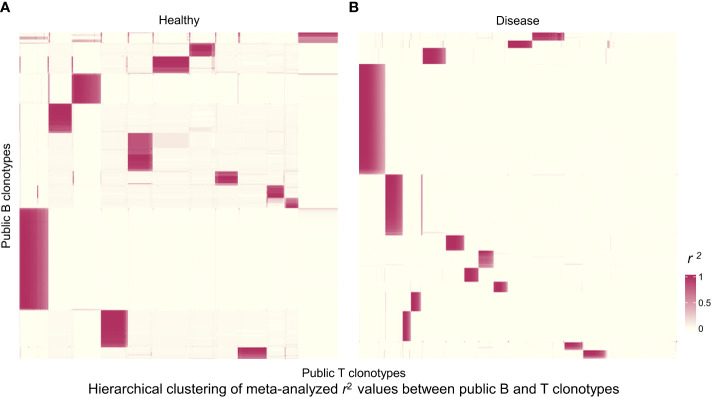
Hierarchically clustered heatmap of correlations between public B and T cell clonotype proportions. **(A)** Heatmap for public clonotypes from healthy individuals. Heatmap displays *r* ^2^ values for 1,244 B cell clonotypes and 4,084 T cell clonotypes. Each rectangle represents a cluster of B and T cell clonotypes analyzed for sequence motifs and amino acid physical properties. **(B)** Heatmap for public clonotypes from individuals with disease. Heatmap displays *r* ^2^ values for 859 B cell clonotypes and 4,459 T cell clonotypes.

We next wanted to broaden our analysis to the entire pool of public clonotypes in all 4 studies and look for any suggestive structure in the correlations between B and T cell clonotype proportions. First, we pooled all public clonotypes from 4 datasets of healthy individuals (2,024 public B cell clonotypes and 27,237 public T cell clonotypes) and calculated the proportion of each public clonotype in each individual’s receptor repertoire. After calculating the clonal proportions for 2,024 B and 27,237 T cell clonotypes for each individual, and quantifying the correlation between all public B and T cell clonotype pairs (2,024×27,237 pairs, where correlation coefficients from individual studies were meta-analyzed), we generated a heatmap of public B and T cell clonotype proportional correlations, where the clonotypes were hierarchically clustered based on the correlations. This resulted in distinct islands of highly correlated B and T cell clonotypes that could be visually appreciated (**Figure 4A**). These islands thus suggested clusters of B and T cell clonotypes that may have an increased likelihood of coexistence in an individual.

We selected the B and T cell clonotypes belonging to the clusters, grouping 61% of all public B cell clonotypes and 15% of all public T cell clonotypes into nonoverlapping clusters. We examined the clonotypes belonging to this groups for any defining or unusual characteristics by comparing their amino acid sequences to those belonging to the rest of the pool of public clonotypes (see **Methods**). Several of the selected B and T cell clonotype clusters contained distinct motifs (selection is shown in **Table 2**).

**Table 2:** Motifs discovered from clusters of healthy and DA B and T cell clonotypes.

B/TCR	Healthy/DA	Sequence logo	Pr in group	Pr outside group	Enrichment ratio	*P*-value
TCR	DA	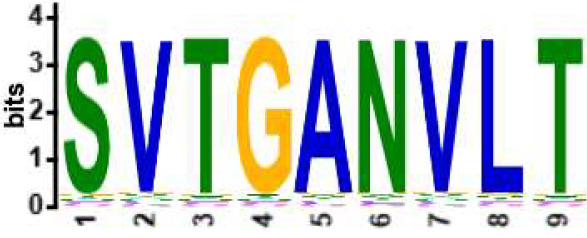	11 /316	0 /24170	N/A	1.6x10^-4^
TCR	DA	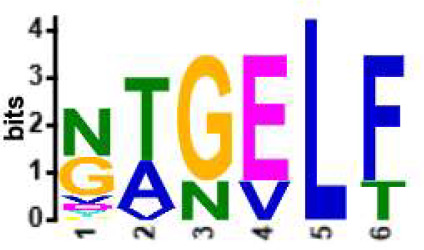	42 /455	126 /24031	17.61	3.7x10^-4^
TCR	DA	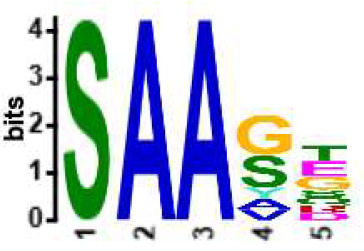	12 /536	2 /23950	268.10	4.3x10^-4^
TCR	DA	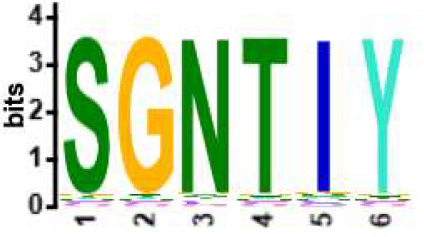	11 /320	56 /24166	14.83	3.4x10^-2^
TCR	Healthy	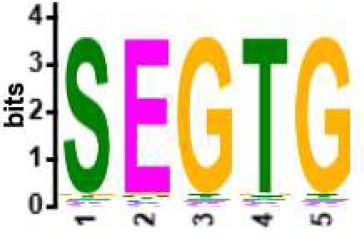	9 /323	13 /33103	70.95	2.1x10^-2^
BCR	DA	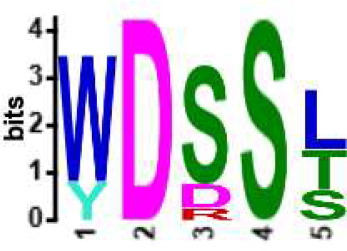	13 /44	0 /2118	N/A	9.2x10^-23^
BCR	DA	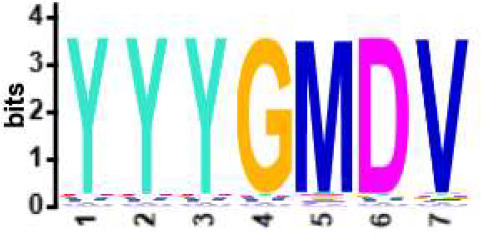	5 /21	0 /2141	N/A	8.0x10^-11^
BCR	DA	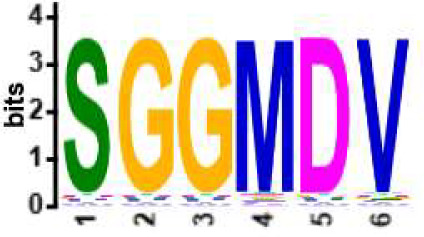	5 /22	0 /2140	N/A	1.1x10^-10^
BCR	DA	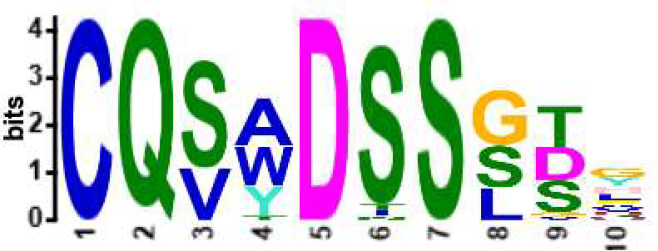	47 /53	175 /5453	27.63	1.3x10^-3^
BCR	Healthy	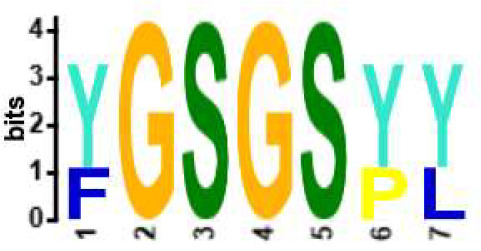	4 /54	0 /33368	N/A	2.9x10^-3^

Pr, proportion. DA, disease-associated.

NA, not available.

**Figure 5:**
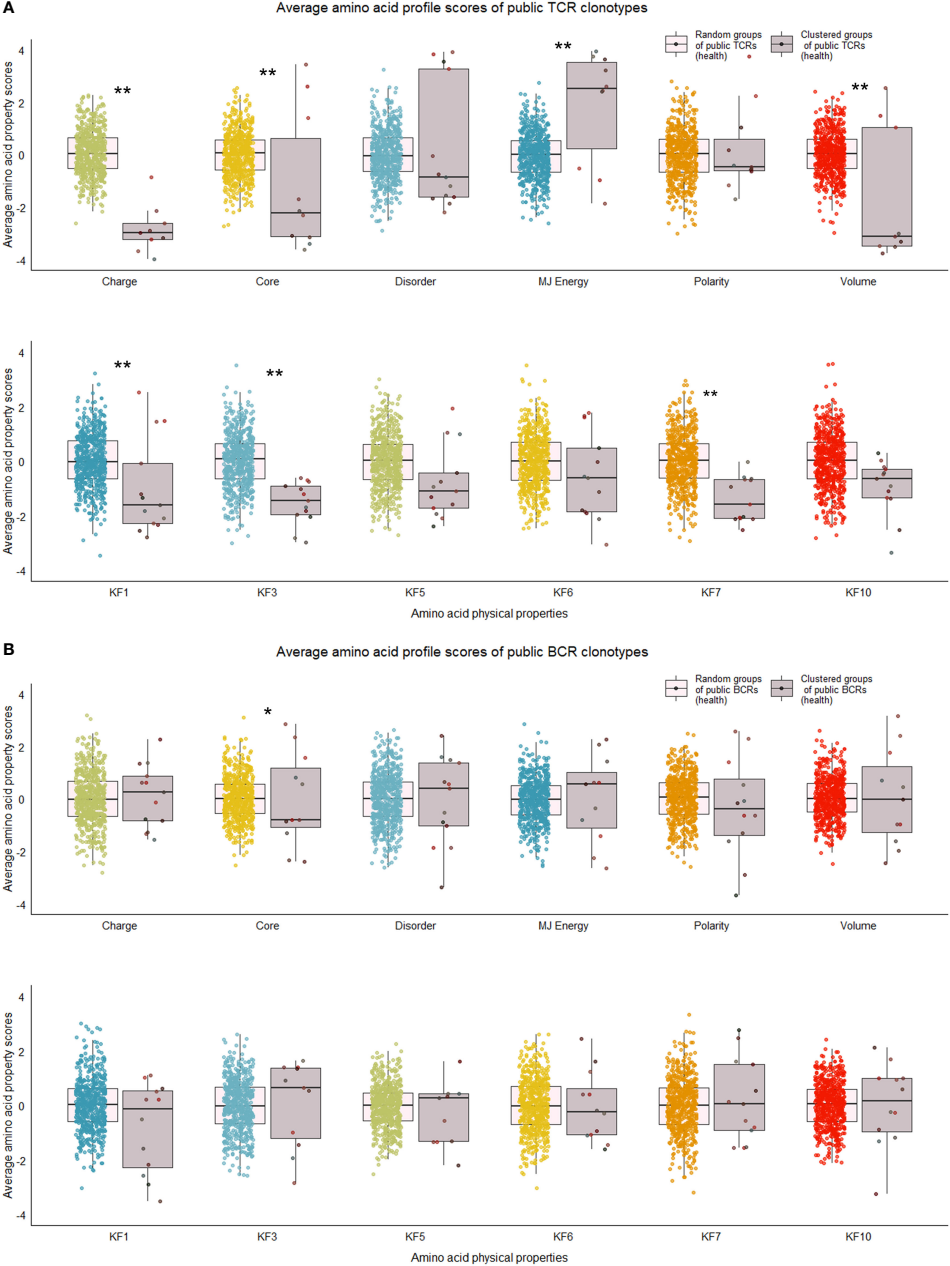
Distribution of average amino acid property scores for selected clusters of public BCR and TCR amino acid sequences compared to random groups of public BCR and TCR amino acid sequences from healthy immune repertoires. **(A)** Boxplots of scaled average amino acid physical property scores of TCR amino acid sequences from selected clusters (mauve colors) compared to those of randomly selected T cell clonotype groups (rainbow colors) of healthy individuals. **(B)** Boxplots of scaled average amino acid physical property scores of BCR amino acid sequences from selected clusters (mauve colors) compared to those of randomly selected B cell clonotype groups (rainbow colors) of healthy individuals. Dots represent averages of each cluster or random group. *P*-values are from Kolmogorov-Smirnov tests for difference of two distributions, i.e. averages of random groups of amino acid sequences and averages of clustered clonotype sequences. ** *P*-value< 0.005. *P-value< 0.05. KF kidera factors (KF1 helix/bend preference, KF3 extended structure preference, KF5 double-bend preference, KF6 partial specific volume, KF7 flat extended preference, KF10 surrounding hydrophobicity).

Our next analyses focused on whether sequence characteristics of clustered clonotypes were different from what could be expected based on the entire pool of public clonotypes. We calculated average values for randomly selected clonotypes from the entire public clonotype pool. Random selection was repeated 10,000 times to form a reference distribution of average amino acid physical property values. We then compared the cluster averages with averages of 10,000 random groups. T cell clonotypes belonging to the cluster showed distinct distributions of average amino acid properties such as charge, and Kidera factors 3 (extended structure preference) and 7 (flat extended preference, **Figure 5A**), meaning that T cell clonotypes belonging to clusters had lower charge, and were less likely to prefer extended and flat extended structures. B cell clonotypes did not show similar distinct distributions of amino acid properties (**Figure 5B**). Clustered T cell clonotypes also showed decreased within-group average pairwise Levenshtein distance (LD) values (**Figure 7A**), suggesting increased amino acid sequence similarity within clusters. These findings show that grouping clonotypes, especially T cell clonotypes, based on correlations of proportions between B and T cell clonotype can reveal functional groups of similar clonotypes that share certain sequence features such as extended structure preference and negative charge.

**Figure 6:**
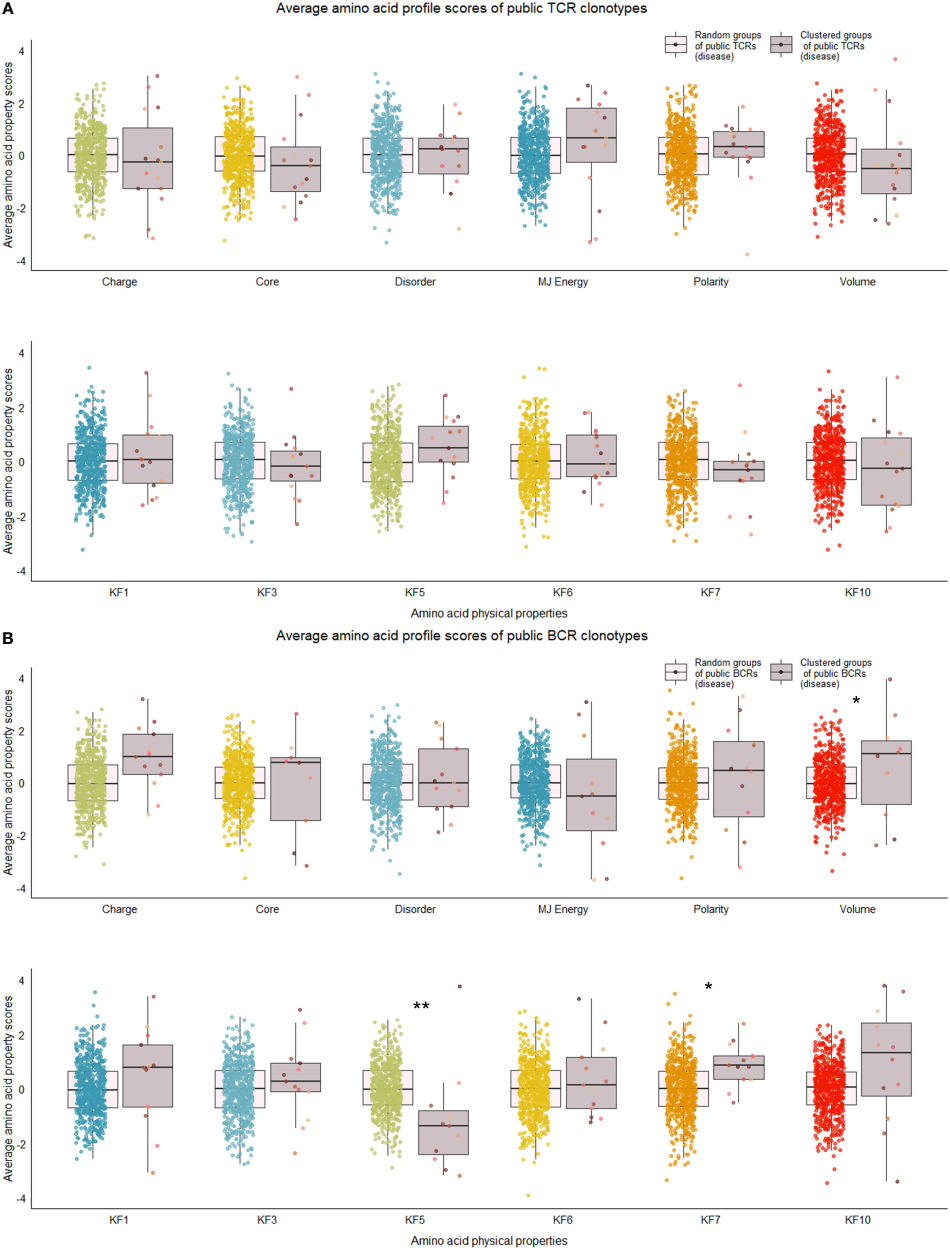
Distribution of average amino acid property scores for selected clusters of public BCR and TCR amino acid sequences compared to random groups of public BCR and TCR amino acid sequences from disease-associated immune repertoires. **(A)** Boxplots of scaled average amino acid physical property scores of TCR amino acid sequences from selected clusters (mauve colors) compared to those of randomly selected T cell clonotype groups (rainbow colors) of individuals with disease. **(B)** Boxplots of scaled average amino acid physical property scores of BCR amino acid sequences from selected clusters (mauve colors) compared to those of randomly selected B cell clonotype groups (rainbow colors) of individuals with disease. Dots represent averages of each cluster or random group. *P*-values are from Kolmogorov-Smirnov tests for difference of two distributions, i.e. averages of random groups of amino acid sequences and averages of clustered clonotype sequences. **P-value< 0.005. * *P*-value< 0.05. KF kidera factors (KF1 helix/bend preference, KF3 extended structure preference, KF5 double-bend preference, KF6 partial specific volume, KF7 flat extended preference, KF10 surrounding hydrophobicity).

**Figure 7:**
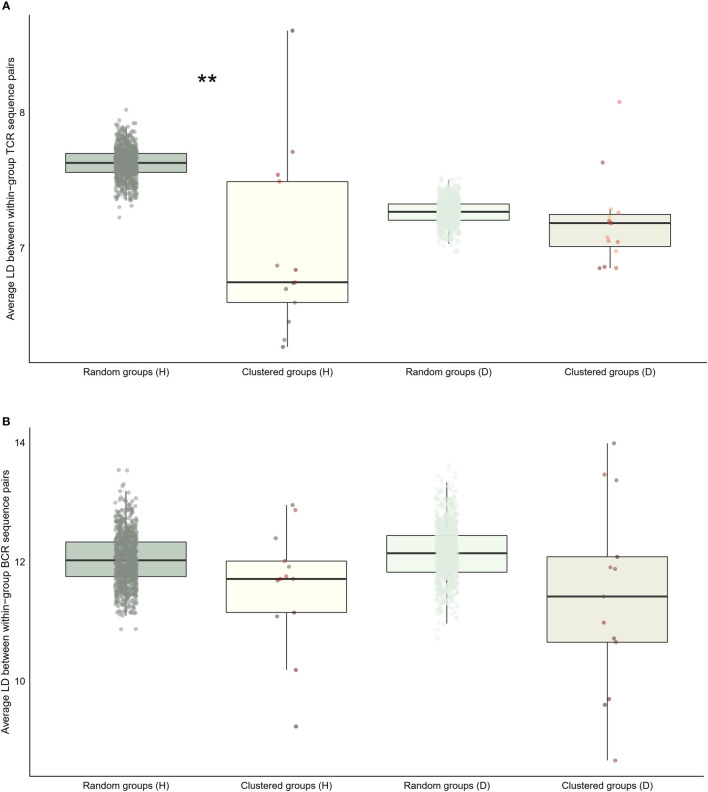
Distribution of average within-group pairwise Levenshtein distances for selected clusters of public BCR and TCR amino acid sequences compared to random groups of public BCR and TCR amino acid sequences. **(A)** Boxplot of averages of Levenshtein distance (LD) between TCR amino acid sequence pairs from healthy (H), and disease (D) clusters compared with averages of LD between TCR amino acid sequence pairs from randomly selected public T cell clonotype groups **(B)** Boxplot of averages of LD between BCR amino acid sequence pairs from healthy (H), and disease (D) clusters compared with averages of LD between BCR amino acid sequence pairs from randomly selected public B cell clonotype groups. Dots represent averages of each cluster or random group. *P*-values are from Kolmogorov-Smirnov tests for difference of two distributions, i.e. averages of random groups of amino acid sequences and averages of clustered clonotype sequences. ** *P*-value< 0.005. *P-value< 0.05.

We repeated the analysis for DA BCR and TCR repertoires. Clusters were discerned from a similar heatmap drawn from 2,179 public B cell clonotypes and 24,399 public T cell clonotypes pooled from 3 DA datasets, clustering 39% of B cell clonotypes and 13% all public T cell clonotypes into nonoverlapping clusters (**Figure 4B**). Several distinct motifs were also discovered in disease B and T cell clonotype clusters (**Table 2**). In contrast to clonotype clusters from healthy individuals, clustered DA T cell clonotypes did not show distinct distributions of average amino acid property scores (**Figure 6A**) compared to the randomly sampled clonotypes. However, clustered DA B cell clonotypes showed distinct distributions of Kidera factors 5 (double bend preference) and 7 (flat extended preference, **Figure 6B**). Average pairwise LD was marginally lower in disease for T cell clonotype groups compared to random groups (**Figure 7A**). Strikingly, random groups of DA public T cell clonotypes showed an already decreased average pair-wise LD compared to healthy public T cell clonotypes, indicating an increased sequence similarity and decreased sequence diversity of DA T cell clonotypes overall (**Figure 7A**). Average LD of random sets of public B cell clonotypes in disease, however, were not decreased compared to healthy (**Figure 7B**). This seems to indicate decreased diversity of public T cell clonotypes in disease, despite a preserved diversity of public B cell clonotypes, as measured by average within group LD, resulting in disease T cell clonotype clusters that do not have the highly distinguishing features of healthy T cell clonotype clusters. A loss of such ‘meaningful’ T cell clonotype clustering could be a feature of disease status, where B and T cell proportional correlation is engendered through different mechanisms from those in health.

### 3.5 T cell clonotype clusters of healthy individuals have increased proportions of sequences belonging to public databases

**Figure 8:**
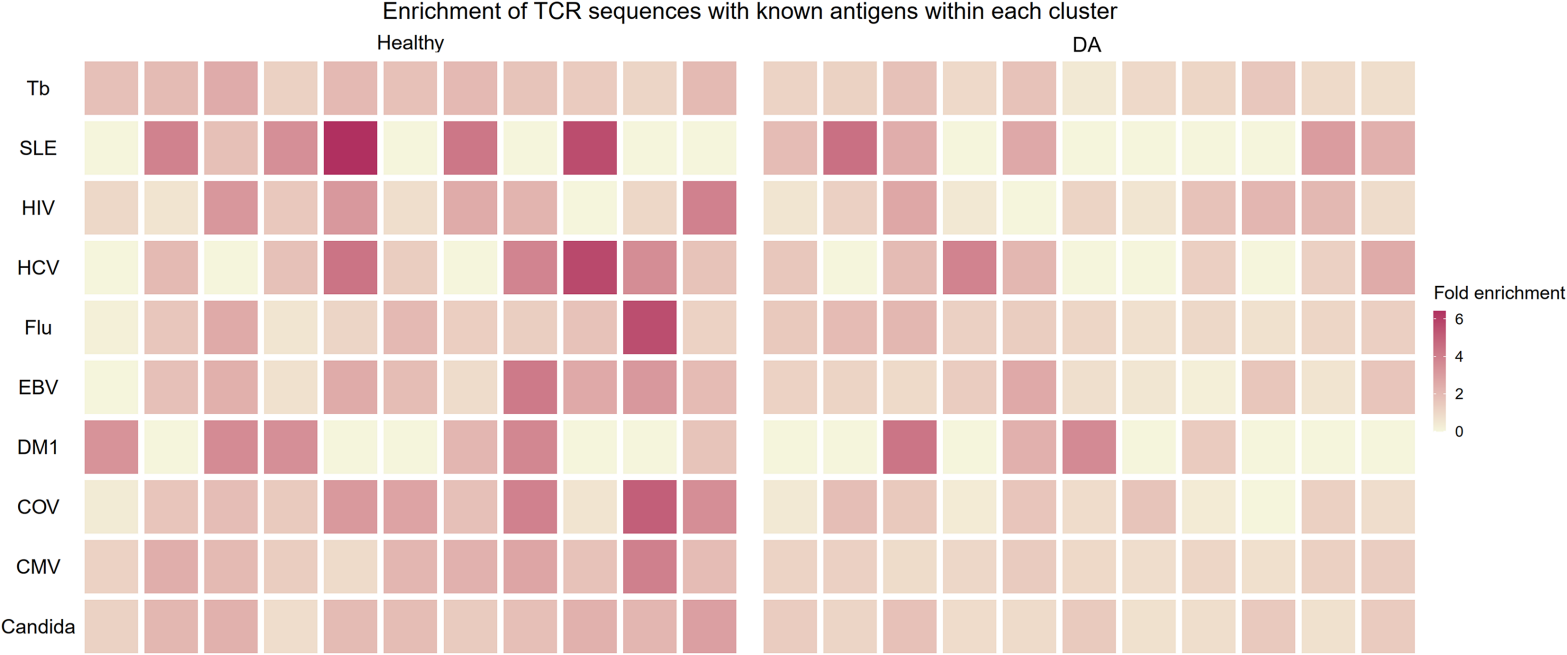
Heatmap showing enrichment of TCR sequences with known antigens within T cell clonotype clusters from proportional correlation-based clustering. DA, disease-associated CMV cytomegalovirus COV SARS-CoV-2 EBV Epstein-Barr virus Flu Influenza virus HCV hepatitis C virus HIV human immunodeficiency virus MTb *Mycobacterium tuberculosis* SLE systemic lupus erythematosus T1DM diabetes mellitus type 1.

In an effort to judge whether the B and T correlation-based grouping of clonotypes discovered clusters of functional importance, we utilized a public database of TCR sequences with known antigens to see whether the clusters contained antigen-specific clonotypes. We restricted our analysis to top ten largest antigen categories: candida, cytomegalovirus (CMV), SARS-CoV-2, diabetes mellitus type 1, Epstein-Barr virus (EBV), human immunodeficiency virus (HIV), hepatitis C virus (HCV), influenza virus, systemic lupus erythematosus (SLE), and *Mycobacterium tuberculosis*. For each antigen category, we calculated the proportional enrichment of matching antigen-specific sequences by comparing these fractions to underlying proportions of antigen-specific sequences in the total pooled public T cell clonotypes from healthy and DA datasets. We found that several healthy T cell clonotype clusters were enriched up to 6-fold for public receptor sequences specific to the influenza virus, HCV, SLE, and the SARS-CoV-2 (**Figure 8**), signaling a selective concentration of antigen specificity in different clusters. This lends further support to the idea that groups of B and T cell clonotypes that tend to be found together within the same individual represent functionally connected clonotypes.

This proportional enrichment was present to a lesser extent in T cell clonotype clusters from individuals with disease (up to 4-fold, **Figure 8**). One possible explanation to account for this finding is that the specificity of clonotypes in the DA clusters may be enriched in a more diverse set of antigens that are disease-related.

### 3.6 Validation of primary findings using a replication dataset

**Figure 9:**
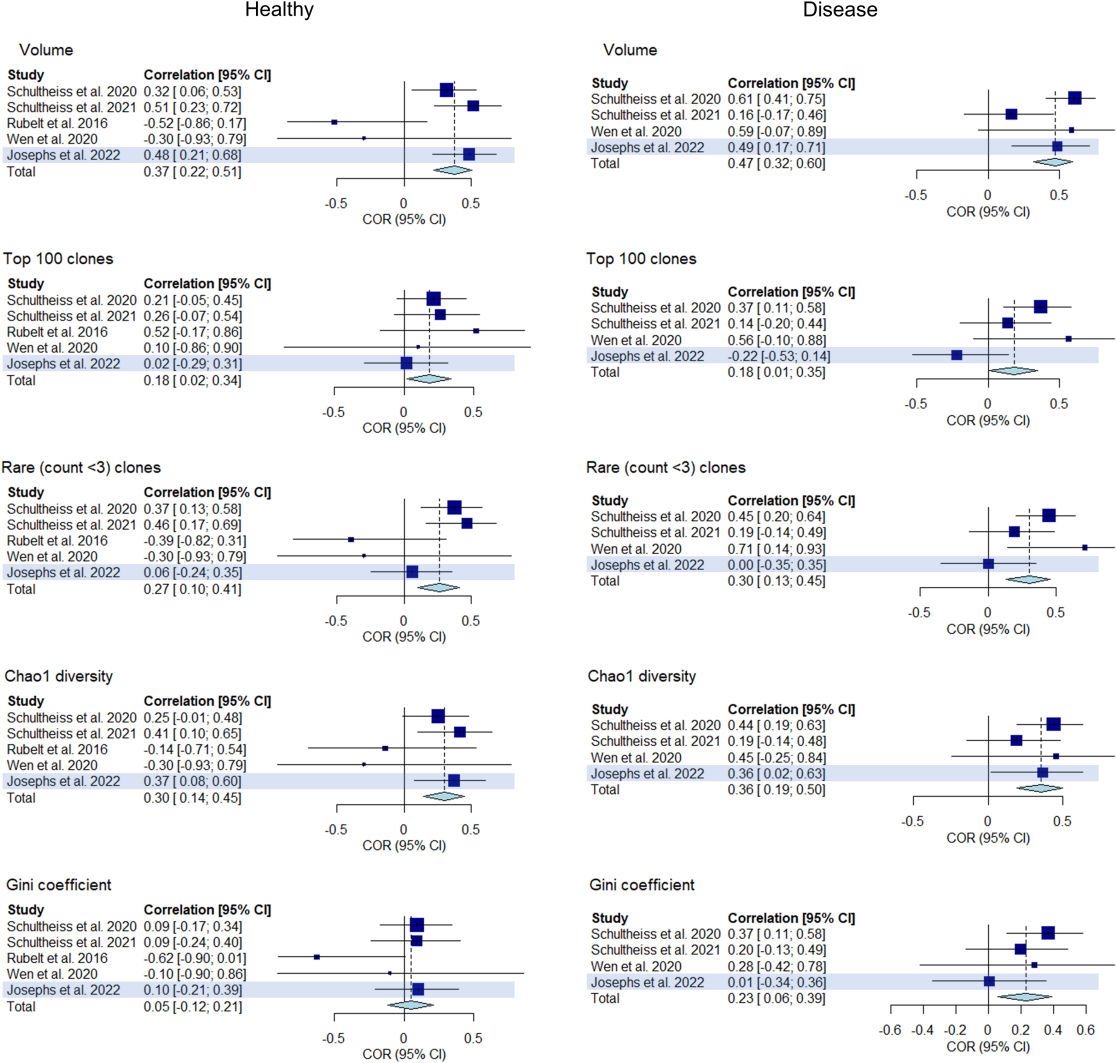
Forest plot of meta-analyses of clonality and diversity metrics with the replication study (using Josephs et al., 2022) included. Meta-analysis results of the correlations between clonality and diversity summary statistics derived from individual BCR and TCR repertoires from each study including the replication study (highlighted) are presented in a forest plot. Size of the squares indicate study weights that are proportional to sample size. Effect sizes are from a common effect model.

In this part of the analysis, we sought to replicate our primary findings by repeating our analysis in a step-by-step manner using an replication dataset of immune repertoires from 43 healthy controls and 32 COVID-19 patients. We measured the correlations between clonality and diversity summary statistics of each individual’s BCR and TCR repertoires which were extracted as detailed previously. The results are presented in **Figure 9** as well as in **Supplementary Tables 1, 2**. The addition of the replication dataset in the meta-analysis led to greater confidence in the correlations of clonal volume (*r* change: 0.33 to 0.37, *P* value change: 0.008 to 7.9e-5) and Chao1 diversity (*r* change: 0.28 to 0.30, *P* value change: 0.03 to 4.2e-3) in the healthy. Similarly, in disease, addition of the replication dataset led to lower *P* values for correlations between clonal volume and Chao1 diversity (*P* value change: 2.0e-5 to 2.9e-7, 1.6e-3 to 6.8e-4, respectively). For network statistics from the replication dataset, two metrics derived from downsampled repertoires to a size of 2000 showed negative correlations in the healthy (average degree and density, **Supplementary Table 2**), although this should be interpreted with caution since network metrics have been shown to be affected by downsampling size.

Finally, we sought to gauge the significance of the paired clusters of public B and T clonotypes by looking at the presence of each clustered clonotype within individuals in the replication dataset. We performed a regression approach (see **Methods**) to assess whether the fractions of BCRs that matched to B cell clonotype clusters can explain the fractions of TCRs that matched to the identical T cell clonotype clusters. We obtained a significant result (regression estimate = 0.125, *P*-value = 1.05e-5) for clusters defined from healthy BCR and TCR repertoires, indicating that the correlations between B and T clonotypes in the clusters defined by the main analysis were also observed in healthy subjects of the replication dataset. This regression estimate value was outside of the distribution of estimates seen in 100 iterations of randomly chosen B and T cell clonotype clusters (**Supplementary Figure 1**), denoting that an empirically assessed p-value was also <0.01. This result suggests that when an individual has many BCRs belonging to one cluster, he or she likely has many TCRs belonging to the paired cluster of T cell clonotypes. The same trend, however, was not observed in individuals with disease, suggesting that the clusters may not translate across different disease activities and states, as the main analysis consisted of two diseases while the replication analysis was fixed to a single disease (COVID-19).

## 4 Discussion

We have explored BCR and TCR together to search for any shared features that shed light on the intertwined workings of the adaptive immune system. In support of our hypothesis that similarities BCR and TCRs result from shared immune history and cellular machinery, we show that several key repertoire features such as clonal volume and species diversity covary in an individual’s BCR and TCR. We observed an increased tendency for an individual’s BCR and TCR repertoire to share clonal architecture and diversity characteristics in disease states. We also found an increased presence of highly correlated B and T cell clonotypes in the memory B and CD4+ T cell subsets of BCR and TCR. Finally, we identified nonoverlapping clusters of public B and T cell clonotypes that tend to be co-present in an individual. B and T cell clonotypes belonging to these clusters, especially the T cell clonotypes from healthy clusters, tended to have increased sequence similarity. T cell clonotype sequences within healthy clusters also had lower charge and a higher preference for flat, extended structures, as well as an enrichment of sequences with specific known antigens.

In our study, we looked at the repertoire summary statistics of both healthy and individuals with disease. While most of the correlations were weak to moderate, we recognized that there were differences in the pattern of correlations. Three categories of summary statistics including clonality metrics (hyperexpanded and top clone proportions) and diversity metrics (Gini-Simpson index and inverse Gini index) were more tightly correlated in the DA group, although the same trend was not seen in the replication dataset. These results may suggest that certain disease states are characterized by ‘hypersynchronized’ BCR and TCR repertoire clonal structures, where an ongoing disease process exerts pressure on both BCR and TCR repertoires to undergo clonal expansion and mobilize to pathogens and disease signals.

We also found that a surprisingly large proportion of public T cell clonotypes cluster with groups of public B cell clonotypes. A T cell receptor bias that leads to the unexplained large presence of public receptor sequences has been observed previously (29, 30). How such a bias takes shape in an individual’s immune repertoire is not yet clear. Our findings suggest that a significant part of the bias that results in unexpectedly abundant public clonotypes occurs through a way that affects both B and T cells and also increases the likelihood of certain public B and T cell clonotypes to be co-present in the immune repertoire. As our finding of the enrichment of known antigen-specific TCR sequences in the clustered T cell clonotypes suggests, such bias shaping may occur through continued antigenic stimulation in autoimmune diseases (SLE, T1DM) and chronic infections such as CMV and HIV infection.

Our results point toward a novel way of discovering functionally relevant clusters B and T cell receptor sequences. Although the cell subtype information for the identified clusters of correlated public B and T cell clonotypes were not known, based on the results from the cell subtype-specific analysis, it is plausible to hypothesize that correlation-based grouping of the public repertoire isolates BCR and TCR clusters from memory cell subsets that represent specific portions of the shared immune memory of BCR and TCR repertoires. One strength of our grouping method is that it is solely based on the physiological phenomenon of the BCRs and TCRs being discovered concurrently in an individual, and does not rely on past assumptions about the receptor sequence itself that are used by many other T cell receptor clustering methods (31, 32). Combining BCR and TCR repertoires to group individual clonotypes into different clusters may thus be a natural way to cluster immune receptors to extract additional functional information about the immune repertoire.

Several caveats need to be kept in mind in interpreting the results of this study. First, repertoire metrics can depend on amount of sample used and sequencing depth. Given the large number of B and T cells in the body, it is impossible to sample and sequence all receptor sequences in a single blood draw, and repertoire characteristics vary depending on the amount of sample and sequencing depth used. Correlation of BCR and TCR size, therefore, may be a result of a correlation in the amount of sample and sequencing depth used for immune sequencing. Although it is impossible to completely remove the influence of this potential confounder, we have tried three things to account for its effects; (1) We performed meta-analysis of statistics that can remove study-specific effects to some degree, (2) We performed downsampling of the repertoire to different sizes to measure uncertainty of results dependent on sampling depths, and (3) We used an independent dataset to replicate results.

Our study is also limited by its small sample size, as the majority immune repertoire sequencing studies so far have focused solely on either BCR or TCR repertoires separately and only a limited number of studies to date have sequenced both the BCR and TCR repertoires of an individual. Another factor that could have influenced our results is the heterogeneous sequencing methods employed by the studies included, comprising both cDNA and DNA sequencing, and the limited information we have on the individual subjects included in the study. Factors such as age and gender are known to influence BCR and TCRs, which we could not adjust for in this study due to the limited availability of demographic information of the individual study participants.

This study provides evidence in favor of studying BCR and TCR repertoires together in order to reveal aspects of the immune system that studying both individually may not be able to unveil. Further examination of the defining shared features both BCR and TCR repertoires may reveal hallmarks of a healthy interaction between B and T cells, as is necessary for a robust immune response, and how they are altered by a history of immune exposures and disease.

## Data availability statement

Publicly available datasets were analyzed in this study. This data can be found here: European Nucleotide Archive accession numbers: PRJEB38339, PRJEB37143, McPAS-TCR: http://friedmanlab.weizmann.ac.il/McPAS-TCR/, VDJdb: https://vdjdb.cdr3.net/, iReceptor Gateway: https://gateway.ireceptor.org/.

## Author contributions

SH and Y-WS conceived the research idea and SH initiated the project. SH and JH gathered data and performed data analyses. SL and BH supervised the project. SH wrote the manuscript and BH, JH, and Y-WS revised the manuscript for intellectual content. All authors contributed to the article and approved the submitted version.

## Funding

This research was supported by a grant of the MD-PhD/Medical Scientist Training program through the Korea Health Industry Development Institute (KHIDI), funded by the Ministry of Health & Welfare, Republic of Korea.

## Conflict of interest

BH is the CTO of Genealogy Inc.

The remaining authors declare that the research was conducted in the absence of any commercial or financal relationships that could be constructed as a potential conflict of interest.

## Publisher’s note

All claims expressed in this article are solely those of the authors and do not necessarily represent those of their affiliated organizations, or those of the publisher, the editors and the reviewers. Any product that may be evaluated in this article, or claim that may be made by its manufacturer, is not guaranteed or endorsed by the publisher.

## References

[1] Bradley P Thomas PG. Using T cell receptor repertoires to understand the principles of adaptive immune recognition. Annu Rev Immunol (2019) 37:547–70. doi: 10.1146/annurev-immunol-042718-041757 30699000

[2] DeKosky BJ Ippolito GC Deschner RP Lavinder JJ Wine Y Rawlings BM. High-throughput sequencing of the paired human immunoglobulin heavy and light chain repertoire. Nat Biotechnol (2013) 31:166–9. doi: 10.1038/nbt.2492 PMC3910347 23334449

[3] Freeman JD Warren RL Webb JR Nelson BH Holt RA. Profiling the T-cell receptor beta-chain repertoire by massively parallel sequencing. Genome Res (2009) 19:1817–24. doi: 10.1101/gr.092924.109 PMC2765271 19541912

[4] Johansson G Kaltak M Rımniceanu C Singh AK Lycke J Malmeström C. Ultrasensitive DNA immune repertoire sequencing using unique molecular identifiers. Clin Chem (2020) 66:1228–37. doi: 10.1093/clinchem/hvaa159 32814950

[5] Miho E Yermanos A Weber CR Berger CT Reddy ST Greiff V. Computational strategies for dissecting the high-dimensional complexity of adaptive immune repertoires. Front Immunol (2018) 9:224. doi: 10.3389/fimmu.2018.00224 29515569 PMC5826328

[6] Goronzy JJ Qi Q Olshen RA Weyand CM. High-throughput sequencing insights into T-cell receptor repertoire diversity in aging. Genome Med (2015) 7. doi: 10.1186/s13073-015-0242-3 PMC4652363 26582264

[7] Martin V Wu Y-CB Kipling D Dunn-Walters D. Ageing of the b-cell repertoire. Philos Trans R Soc B: Biol Sci (2015) 370:20140237. doi: 10.1098/rstb.2014.0237 PMC4528414 26194751

[8] Ye B Smerin D Gao Q Kang C Xiong X. High-throughput sequencing of the immune repertoire in oncology: Applications for clinical diagnosis, monitoring, and immunotherapies. Cancer Lett (2018) 416:42–56. doi: 10.1016/j.canlet.2017.12.017 29247824

[9] Hu X Zhang J Wang J Fu J Li T Zheng X. Landscape of b cell immunity and related immune evasion in human cancers. Nat Genet (2019) 51:560–7. doi: 10.1038/s41588-018-0339-x PMC6773274 30742113

[10] Bashford-Rogers RJM Smith KGC Thomas DC. Antibody repertoire analysis in polygenic autoimmune diseases. Immunology (2018) 155:3–17. doi: 10.1111/imm.12927 29574826 PMC6099162

[11] Fu Y Li B Li Y Wang M Yue Y Xu L. A comprehensive immune repertoire study for patients with pulmonary tuberculosis. Mol Genet Genomic Med (2019) 7. doi: 10.1002/mgg3.792 PMC6625341 31173489

[12] Wang P Jin X Zhou W Luo M Xu Z Xu C. Comprehensive analysis of TCR repertoire in COVID-19 using single cell sequencing. Genomics (2021) 113:456–62. doi: 10.1016/j.ygeno.2020.12.036 PMC7833309 33383142

[13] Corrie BD Marthandan N Zimonja B Jaglale J Zhou Y Barr E. iReceptor: A platform for querying and analyzing antibody/B-cell and T-cell receptor repertoire data across federated repositories. Immunol Rev (2018) 284:24–41. doi: 10.1111/imr.12666 29944754 PMC6344122

[14] Linnemann C Heemskerk B Kvistborg P Kluin RJC disease B Chen X. High-throughput identification of antigen-specific TCRs by TCR gene capture. Nat Med (2013) 19:1534–41. doi: 10.1038/nm.3359 24121928

[15] Schultheiß C Paschold L Simnica D Mohme M Willscher E von Wenserski L. Next-generation sequencing of T and b cell receptor repertoires from COVID-19 patients showed signatures associated with severity of disease. Immunity (2020) 53:442–455.e4. doi: 10.1016/j.immuni.2020.06.024 32668194 PMC7324317

[16] Schultheiß C Simnica D Willscher E Oberle A Fanchi L Bonzanni N. Next-generation immunosequencing reveals pathological T-cell architecture in autoimmune hepatitis. Hepatology (2021) 73:1436–48. doi: 10.1002/hep.31473 32692457

[17] Bolotin Poslavsky S Mitrophanov I Shugay M IZ M EV P. MiXCR: software for comprehensive adaptive immunity profiling. Nat Methods (2015) 12:380–1. doi: 10.1038/nmeth.3364 25924071

[18] Rubelt F Bolen CR McGuire HM Heiden JAV Gadala-Maria D Levin M. Individual heritable differences result in unique cell lymphocyte receptor repertoires of naïve and antigen-experienced cells. Nat Commun (2016) 7. doi: 10.1038/ncomms11112 PMC5191574 27005435

[19] Wen W Su W Tang H Le W Zhang X Zheng Y. Immune cell profiling of COVID-19 patients in the recovery stage by single-cell sequencing. Cell Discovery (2020) 6:31. doi: 10.1038/s41421-020-0168-9 32377375 PMC7197635

[20] Joseph M Wu Y Dannebaum R Rubelt F Zlatareva I Lorenc A. Global patterns of antigen receptor repertoire disruption across adaptive immune compartments in COVID-19. PNAS (2022) 119:34. doi: 10.1073/pnas.2201541119 PMC9407655 35943978

[21] Team I. Immunarch: An r package for painless bioinformatics analysis of T-cell and b-cell immune repertoires. (2019). doi: 10.5281/zenodo.3367200

[22] Miho E Roškar R Greiff V Reddy ST. Large-Scale network analysis reveals the sequence space architecture of antibody repertoires. Nat Commun (2019) 10. doi: 10.1038/s41467-019-09278-8 PMC6428871 30899025

[23] Hagberg A Swart P S Chult D. Exploring network structure, dynamics, and function using networkx (2008). Available at: https://www.osti.gov/biblio/960616.

[24] Shugay M Bagaev DV Turchaninova MA Bolotin OV B EV P. VDJtools: Unifying post-analysis of T cell receptor repertoires. PloS Comput Biol (2015) 11:e1004503. doi: 10.1371/journal.pcbi.1004503 26606115 PMC4659587

[25] Bailey TL. STREME: accurate and versatile sequence motif discovery. Bioinformatics (2021) 37:2834–40. doi: 10.1093/bioinformatics/btab203 PMC8479671 33760053

[26] Tickotsky N Sagiv T Prilusky J Shifrut E Friedman N. McPAS-TCR: A manually curated catalogue of pathology-associated T cell receptor sequences. Bioinformatics (2017) 33:2924–9. doi: 10.1093/bioinformatics/btx286 28481982

[27] Bagaev DV Vroomans RMA Samir J Stervbo U Rius C Dolton G. VDJdb in 2019: Database extension, new analysis infrastructure and a T-cell receptor motif compendium. Nucleic Acids Res. (2020) 48:D1057–62. doi: 10.1093/nar/gkz874 PMC6943061 31588507

[28] Benjamini Y Hochberg Y. Controlling the false discovery rate: a practical and powerful approach to multiple hypothesis testing. J R Stat Soc B (1995) 57:289–300. doi: 10.1111/j.2517-6161.1995.tb02031.x

[29] Turner SJ Doherty PC McCluskey J Rossjohn J. Structural determinants of T-cell receptor bias in immunity. Nat Rev Immunol (2006) 6:883–94. doi: 10.1038/nri1977 17110956

[30] Soto C Bombardi RG Kozhevnikov M Sinkovits RS Chen EC Branchizio A. High frequency of shared clonotypes in human T cell receptor repertoires. Cell Rep (2020) 32:107882. doi: 10.1016/j.celrep.2020.107882 32668251 PMC7433715

[31] Huang H Wang C Rubelt F Scriba TJ Davis MM. Analyzing the mycobacterium tuberculosis immune response by T-cell receptor clustering with GLIPH2 and genome-wide antigen screening. Nat Biotechnol (2020) 38:1194–202. doi: 10.1038/s41587-020-0505-4 PMC7541396 32341563

[32] Dash P Fiore-Gartland AJ Hertz T Wang GC Sharma S Souquette A. Quantifiable predictive features define epitope-specific T cell receptor repertoires. Nature (2017) 547:89–93. doi: 10.1038/nature22383 28636592 PMC5616171

