# Supplementary material for: Exploration of shared features of B cell receptor and T cell receptor repertoires reveals distinct clonotype clusters
Source: Front Immunol. 2022 Oct 20;13:1006136. doi: 10.3389/fimmu.2022.1006136 (PMC9632170; doi:10.3389/fimmu.2022.1006136)
Supplement: Supplementary file 1 [file DataSheet_1.docx]

**Exploration of shared features of B cell receptor and T cell receptor repertoires reveals distinct clonotype clusters**

Sang Bin Hong, Yong-Won Shin, Ja Bin Hong, Sang Kun Lee, and Buhm Han

**Supplementary materials**

1. **Supplementary Note**
2. **Calculation of diversity estimators**

**Chao1 estimator**

Chao1 estimator is a nonparametric estimator of species richness that estimates the ‘true’ number of species based on the presence of rare species by the following equation:

$$S_{1}=S_{obs}+\frac{{F_{1}}^{2}}{2 F_{2}}$$

*F_1_*: number of species with a single occurrence

*F_2_*: number of species with two occurrences

**Hill numbers**

The Hill number *^q^D*, or effective number of species, quantifies diversity as follows:

$${}^{q}D= \left( \sum_{i=1}^{S} p_{i}^{q} \right)^{1/(1-q)}$$

*S*: total number of species

*p*_i_: relative abundance of *i*th species

*q*: determines the sensitivity of the measure to relative frequencies

For *q* = 0, *D* simply quantifies species richness.

For *q* = 1, *D* is undefined, but the limit of the equation with $q \to1$ is the exponential of the Shannon’s entropy index (Shannon diversity):

$${}^{1}D= \lim_{q\to1} {}^{q}D=exp\left( -\sum_{i=1}^{S} p_{i}\log p_{i} \right)$$

For *q* = 2, *D* yields Simpson diversity, the inverse of Simpson’s concentration index, as follows:

$${}^{2}{D= \frac{1}{\sum_{i=1}^{S} p_{i}^{2}}}$$

**Gini-Simpson index**

Gini-Simpson index represents the probability that two species taken at random represent different species, also known as the probability of interspecific encounter.

**Gini coefficient**

The Gini coefficient measures the degree of inequality of species frequency along a frequency distribution. The Gini coefficient ranges from 0 to 1, where 0 represents perfect equality, and 1 represents perfect inequality.

1. **Calculation of network metrics**

**Assortativity coefficient**

The assortativity coefficient *r* measures the extent of the similarity of the connections of each node depending on its degree. A perfectly disassortative network (r = -1) refers to a network in which all edges connect to nodes of different degrees, and is seen in more random networks.

**Average degree**

The average degree *k* refers to the average number of edges a node has, and is calculated with the following equation:

$$k= \frac{2L}{N}$$

*L*: total number of edges, *N*: total number of nodes

**Average clustering coefficient**

The clustering coefficient computes the average of clustering coefficients of individual node in the graph, i.e. the number of triangles through the node as a fraction of the possible number of triangles through the node. This measures the tendency of the network to form clusters of connected nodes.

**Density**

The density of a graph is given by the following equation:

$$d= \frac{2L}{N (N-1)}$$

*L*: number of edges, *N*: number of nodes

**Local and global efficiency**

Efficiency of a pair of nodes refers to the inverse of the shortest path between two nodes. The average efficiency between all pairs of nodes in a graph is the global efficiency. The local efficiency is the average global efficiency of subgraphs within the whole graph.

1. **Legends for supplementary tables (presented in supplementary excel file)**

**Supplementary Table 1 Correlation coefficients *r* and *P*-values from correlation of clonality and diversity metrics between BCR and TCR repertoires**

We report the correlation coefficients *r* and respective FDR-corrected *P* values obtained for 10 clonality and diversity metrics for healthy and disease-associated immune repertoires. Tests and downsampling were conducted with the original datasets including studies by Schultheiss et al. 2020, Schultheiss et al. 2021, Rubelt et al. 2016, and Wen et al. 2020. as well as the validation study by Josephs et al. 2022.

**Supplementary Table 2 Spearman rank correlation coefficients r and p-values from correlation testing of network metrics between BCR and TCR repertoires**

We report the correlation coefficients *r* and respective FDR-corrected *P* values obtained for 6 network metrics for healthy and disease-associated immune repertoires. Tests were conducted on downsampled datasets with multiple repertoire sizes.

**Supplementary Table 3 FDR-adjusted P-values for Kolmogorov-Smirnov tests of average amino acid profile scores of clustered clonotype sequences vs random groups of clonotype sequences**

We present the *P*-values from Kolmogorov-Smirnov tests assessing the difference in the distributions of 12 average amino acid quality scores and average pairwise levenshtein distances (LD) for clustered groups of B and T cell clonotypes as compared to random groups of B and T cell clonotypes.

1. **Supplementary Figure**


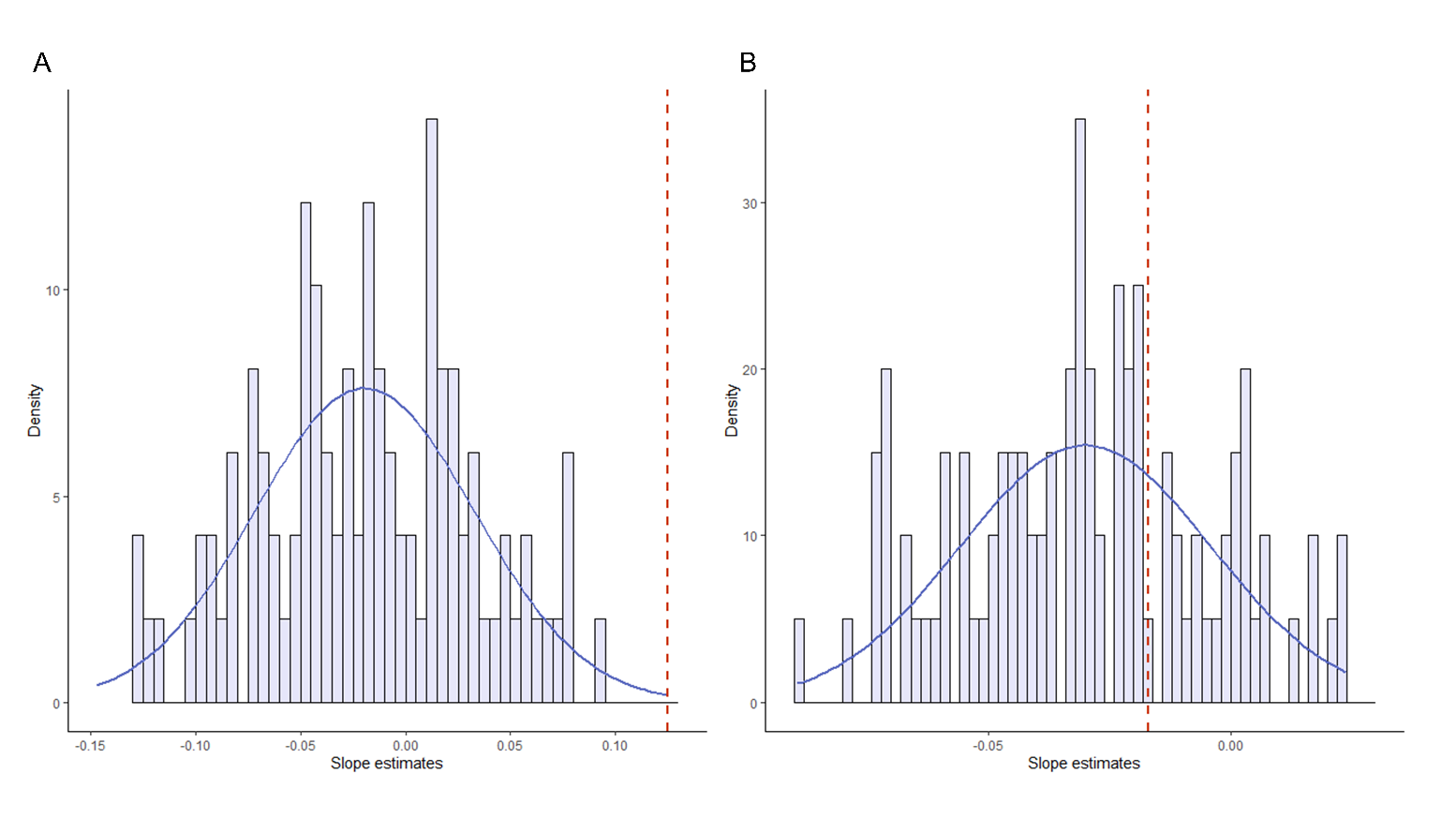


**Supplementary Figure 1 Distribution of slope estimates from the regression model with random groups of public B and T cell clonotypes compared to the slope estimate from the B and T cell clonotype clustered based on correlation of proportions**

**A** Public B and T cell clonotypes were pooled from healthy repertoires, and fractions of each BCR and TCR groups measured in the repertoires of healthy individuals from the separate dataset. Slope estimates roughly follow a normal distribution with a mean at 0. The slope estimate based on clustered B and T cell clonotype groups is above all 100 simulated slope estimates.

**B** Public B and T cell clonotypes were pooled from disease-associated repertoires, and fractions of each BCR and TCR groups measured in the repertoires of individuals with disease from the separate dataset. Slope estimates roughly follow a normal distribution with a mean at 0. The slope estimate based on clustered B and T cell clonotype groups falls within the distribution of 100 simulated slope estimates.

Dashed red line represents the slope estimate of the regression model with fractions calculated from the clustered B and T cell clonotypes.
